# Midodrine in Liver Cirrhosis With Ascites: A Systematic Review and Meta-Analysis

**DOI:** 10.7759/cureus.27483

**Published:** 2022-07-30

**Authors:** Dhan B Shrestha, Pravash Budhathoki, Yub Raj Sedhai, Ram Kaji Baniya, Pearlbiga Karki, Pinky Jha, Gaurab Mainali, Roshan Acharya, Amik Sodhi, Dipen Kadaria

**Affiliations:** 1 Department of Internal Medicine, Mount Sinai Hospital, Chicago, USA; 2 Department of Internal Medicine, Bronxcare Health System, New York, USA; 3 Department of Internal Medicine, Virginia Commonwealth University School of Medicine, Richmond, USA; 4 Department of Internal Medicine, Our Lady of Lake Regional Medical Centre, Louisiana, USA; 5 Department of Internal Medicine, Nepalese Army Institute of Health Sciences, Kathmandu, NPL; 6 Internal Medicine, Cape Fear Valley Hospital, Fayetteville, USA; 7 Internal Medicine, Campbell University School of Osteopathic Medicine, Fayetteville, USA; 8 Department of Pulmonary, Critical Care, and Sleep Medicine, University of Tennessee, Memphis, USA; 9 Department of Internal Medicine, University of Tennessee, Memphis, USA

**Keywords:** meta-analysis, systematic review, ascites, cirrhosis, albumin, midodrine

## Abstract

Ascites is the most common complication of liver cirrhosis. Midodrine is a vasoconstrictor that improves splanchnic and systemic hemodynamics, reduces ascites, and improves clinical outcomes. Here, we aimed to examine the role of midodrine in cirrhosis-related ascites.

Scopus, Embase, PubMed, and PubMed Central databases were searched for relevant randomized controlled trials comparing midodrine with other interventions in patients with cirrhotic ascites on November 25, 2020, using appropriate keywords like “midodrine”, “ascitic cirrhosis”, “peritoneal paracentesis” and suitable Boolean operators. Odds ratio (OR) and mean difference (MD) were used to analyze pool data as appropriate with a 95% confident interval (CI).

A total of 14 studies were included in our analysis including 1199 patients. The addition of midodrine resulted in statistically significant improvement in mean arterial pressure (MAP) (MD, 3.95 mmHg; 95% CI, 1.53-6.36) and MELD (Model for End-Stage Liver Disease) score (MD, -1.27; 95% CI, -2.49 to -0.04) compared to standard medical treatment (SMT). There was also a significant improvement in plasma renin activity and plasma aldosterone concentration. However, there was no significant improvement in mortality or serum creatinine compared to SMT. In addition, there was no statistically significant improvement in MAP, plasma renin activity, plasma aldosterone concentration, MELD score, overall mortality, and paracentesis-induced circulatory dysfunction comparing midodrine with albumin.

Midodrine alone leads to significant improvement in various clinical parameters in patients with cirrhotic ascites compared to standard medical care. At the same time, it was found to be non-inferior to albumin. Therefore, further well-designed studies need to be carried out on midodrine in addition to albumin for optimal clinical benefits among patients with ascites due to cirrhosis.

## Introduction and background

Ascites is one of the most common and serious complications of liver cirrhosis [1]. Ascites is managed with diuretics and sodium restriction. Ascites that does not reduce or that occurs shortly after therapeutic paracentesis despite sodium restriction and diuretic treatment is called refractory ascites [2]. Therapeutic paracentesis, combined with the expansion of plasma volume using albumin, is an effective and safe procedure with fewer risks than diuretic therapy in such cases [1]. Albumin, however, is expensive and, may have some risk of disease transmission; its use is thus controversial in some countries [1,3,4]. Peripheral arterial vasodilation has been hypothesized to be the critical factor in the pathogenesis of functional renal abnormalities in patients with cirrhosis [5]. Vasoconstrictor administration may decrease arteriolar vasodilation caused by paracentesis and prevent complications associated with a decrease in the effective arterial blood volume. Midodrine, an alpha-1 agonist directly acting on peripheral alpha-receptors, is a vasoconstrictor and is available as a cheap oral formulation. It has been commonly used to treat orthostatic hypotension and multiple secondary hypotensive disorders [6-8]. Recently, a single-dose administration of midodrine has been shown to substantially improve the systemic and renal hemodynamics of ascites in non-azotemic cirrhotic patients [7]. However, clinical trials evaluating Midodrine have provided inconclusive findings in patients with liver cirrhosis-related ascites, irrespective of the refractory status of the ascites [9].

We aimed to conduct a systematic review and meta-analysis to assess the effectiveness of midodrine in reducing mortality, improving response rates in patients with ascitic cirrhosis undergoing peritoneal paracentesis/drainage, assessment of MELD (Model For End-Stage Liver Disease), plasma renin, aldosterone, and creatinine.

## Review

Methods

Protocol

PRISMA (Preferred Reporting Items for Systematic Reviews and Meta-Analyses) guideline was followed to carry out our systematic review and meta-analysis and is registered in PROSPERO (CRD42020222872) [10].

Eligibility Criteria

We included randomized controlled trials comparing midodrine with control intervention (e.g., placebo, sodium restriction, diuretic treatment, and therapeutic paracentesis) or an active intervention (e.g., different drug) in patients with cirrhotic ascites; and complete data for at least one primary end-point was reported. Studies like editorials, commentary, viewpoint, case reports and series, observational studies, and studies on animal or cell lines were excluded. In addition, articles with no proper data on midodrine on cirrhotic ascites and lacking adequate data of interest were excluded.

Search Strategy

Scopus, Embase, PubMed, and PubMed Central were used to search relevant articles till November 25, 2020, using appropriate keywords like “midodrine”, “ascitic cirrhosis”, and “peritoneal paracentesis,” and suitable Boolean operators. The detailed search strategy is mentioned in the supplementary file.

Study Selection

Two reviewers (PJ and GM) independently screened the title and abstract of imported studies, and any arising conflict was solved by the third reviewer (PK). A full-text review was done independently by PJ and PK. Data were extracted for both quantitative and qualitative synthesis. The conflicts were resolved by taking the third reviewer's opinion (GM). All the screening was done with the help of Covidence [11].

Data Extraction

A standardized form was designed in Microsoft Word to extract pertinent data, including study authors, study details, quality, and endpoints. The endpoints for meta-analysis were the effect of midodrine on short-term mortality within the first three months, paracentesis-induced circulatory dysfunction, mean arterial pressure, MELD scores, serum creatinine, plasma renin, and aldosterone in cirrhotic ascites [12].

Study Quality

**Figure 1:**
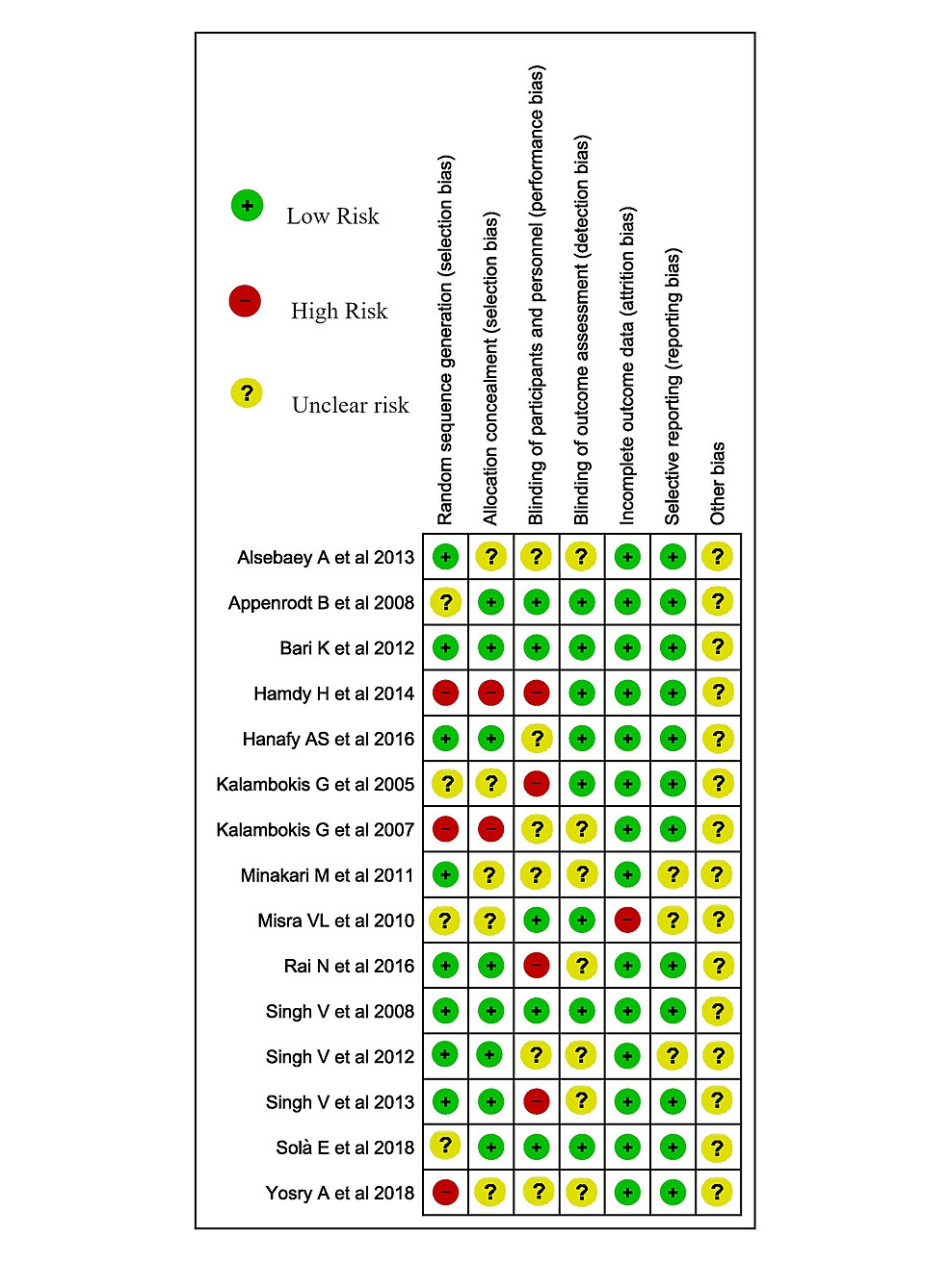
Risk of Bias assessment of included RCTs Included studies are reference nos. [1, 3, 4, 6-8, 14-22]

The quality of individual articles was evaluated using the Cochrane ROB (Risk of Bias) 2.0 for RCTs [13]. The risk of bias was assessed (Figure 1). Two of the authors independently assessed the design of each study, and the number of patients in outcomes including short-term mortality, paracentesis-induced circulatory dysfunction, serum creatinine, plasma renin, plasma aldosterone, and MELD scores. Third-person (among authors) resolved the disagreement.

Data Analysis

Data were analyzed using RevMan v5.4 (https://training.cochrane.org/). Odds ratio (OR) was used for outcomes like short-term mortality and paracentesis-induced circulatory dysfunction (PICD). Heterogeneity was measured by the I² test among the included studies. For data synthesis, a qualitative approach was planned. The handling of data and combining results of the studies was done using OR and using the random or fixed effect model based on heterogeneities. We analyzed the mean difference among the two groups for mean arterial pressure, MELD scores, plasma renin, plasma aldosterone, and serum creatinine level.

Sensitivity Analysis

Subgroup analysis was done within the respective outcomes contrasting albumin-based control and other treatments as a control.

Publication Bias

Publication bias of the included studies was assessed and presented using Funnel plots.

Results

**Figure 2:**
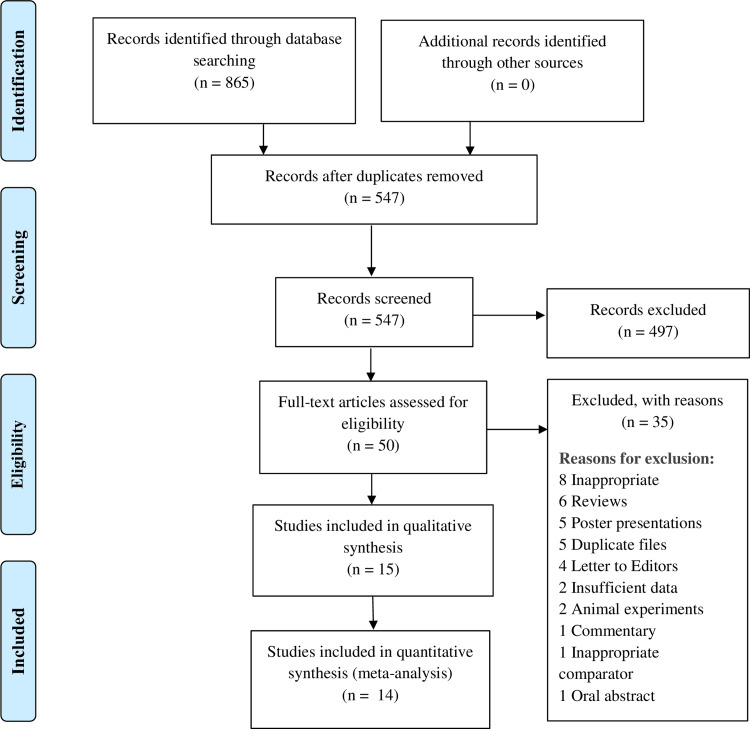
PRISMA Flow Diagram PRISMA= Preferred Reporting Items for Systematic Reviews and Meta-Analyses

We identified a total of 865 studies through a thorough database search. A total of 318 duplicates were removed, and we screened the title and abstracts of 547 studies. After excluding 497 studies, we assessed the full text of 50 studies, and 30 studies were excluded for definite reasons (Figure 2). Therefore, 15 remaining studies were included in our qualitative analysis.

Narrative summary

Qualitative Analysis

We included 15 studies in our qualitative analysis presented in Table 1. Basic details of included studies are shown in the supplementary file. Narrative summary of included studies is shown in Table 1 [1,3,4,6-8,14-22].

**Table 1:** Narrative summary of included studies Abbreviations: ALT= Alanine transaminase, AST= Aspartate aminotransferase, BUN= Blood urea nitrogen , C= Control group, CO(L/min)= Cardiac output, ClCre= Creatinine clearance, CTP score= Child-Turcotte-Pugh score, EF= Ejection fraction, F= Female, GCRC= General Clinical Research Center, GFR(ml/min)= Glomerular filtration rate, HBV= Hepatitis B virus, HCC= Hepatocellular carcinoma, HCV= Hepatitis C virus, HE= Hepatic encephalopathy, HR= Heart rate, HRS= Hepatorenal syndrome, INR= International normalized ratio,  IQR= Interquartile range, IV= Intravenous, M= Male, MAP (mmHg )= Mean Arterial Pressure, MELD score= Model End Stage Liver Disease score, N= Total number, PAC/PA(pg/mL)= Plasma aldosterone concentration, PICD/ PCD= Paracentesis Induced Circulatory Dysfunction, PO= Per-oral, PRA(ng/mL/h)= Plasma renin activity, SAAG= serum-ascites albumin gradient, SBP= Spontaneous bacterial peritonitis,  SMT= Standard medical therapy,  S-Na(mEq/L)= Serum Sodium, SVR(dynes/s/cm5)= Systemic vascular resistance, T= Treatment group,  UGI= Upper gastrointestinal, U-Na(mEq/24 h)= Urinary sodium, UV= Urinary volume Alsebaey et al. (2013) [14]; Appenrodt et al. (2008) [1]; Yosry et al. (2018) [4]; Bari et al. (2012) [15]; Hamdy et al. (2014) [16]; Hanafy et al. (2016) [6]; Kalambokis et al. (2005) [8]; Kalambokis et al. (2007) [17]; Minakari et al. (2011) [18]; Misra et al. (2010) [7]; Rai et al. (2016) [19]; Singh et al. (2008) [3]; Singh et al. (2012) [20]; Singh et al. (2013) [21]; Solà et al. (2018) [22]

Study ID	Particulars	Intervention group	Comparator group
Alsebaey et al. (2013)	Year	2013	
	Study design	RCT	
	Total participants	50	
	Description	Oral midodrine (5–10 mg three times daily)	Standard-dose albumin (6 g/l ascetic fluid removed) Others intravenous terlipressin (3 mg), intravenous Hydroxyethyl Starch(HES) (8 g/l ascetic fluid removed), Low-dose albumin (2 g/l ascetic fluid removed )
	Population characteristics		
	Participants	25	25
	Male (number/total)	18/25	9/25
	Female (number/total)	7/25	16/25
	Weight (Kg)	82.04 ± 10.49	87.08 ± 14.18
	Baseline Values		
	MELD score	13.68 ± 4.17	15.28 ± 4.11
	MAP(mmHg)	77.44 ± 6.54	77.58 ± 5.81
	Serum creatinine(mg/dL)	0.92 ± 0.37	0.85 ±0.36
	Plasma renin (mU/ml)	162.38 ± 91.00	165.93± 95.34
	Aldosterone(pg/ml)	797.66 ±755.07	837.50±899.48
	Outcome		
	Change in Values on Day 6		
	ΔMAP (mmHg)	0.00 ± 7.65	– 1.19 ± 6.09
	ΔMELD score	0.04 ± 2.24	0.12 ± 1.59
	ΔSerum creatinine(mg/dL)	0.02 ± 0.23	0.06 ±0.29
	ΔUrine output (ml/min)	292.00 ± 400.96	468.00± 324
	ΔPRA (µU/ml)	30.75 ± 85.07	26.28 ± 30.20
	ΔAldosterone(pg/ml)	-26.60±633.89	9.84±828.46
	Risk of development of paracentesis-induced circulatory dysfunction PICD		
	Positive(number/total)	5/25	3/25
	Negative(number/total)	20/25	22/25
Appenrodt et al. (2008)	Year	2008	
	Study design	RCT	
	Total participants	24	
	Description	Midodrine (12.5 mg post-paracentesis every 8 h for 2 days, six doses each) after the end of paracentesis	Albumin(8 g/L of removed ascites) with placebo pills
	Population characteristics		
	Participants	11	13
	Male(number/total)	7/11	9/13
	Female(number/total)	4/11	4/13
	AGE mean	52 (48;61)	60 (50;63)
	Weight (kg)	67±11	69±13
	Baseline Values		
	Volume of ascites removed (l)	7 (5.7; 10)	5.5(5;7.7)
	MELD score	11(8;14)	11 (6;17)
	MAP (mmHg)	77 (70;79)	76 (63;82)
	Serum creatinine(mg/dL)	0.98 (0.78;1.16)	1 (0.88;1.12)
	Creatinine clearance(ml/min)	66 (25.5;80)	63.5 (39.8;85.3)
	S-Na(mmol/L)	131 (128;133)	129 (125;131)
	Plasma renin (mU/ml)	677.5 (179.7;2016.3)	385(173;2529)
	PAC (pg/mL)	858 (743.6;1446	911(437;1816.5)
	Outcome		
	Median values with IQR On day 6		
	MAP (mmHg)	80 (62;91)	81 (74;83)
	Serum creatinine(mg/dL)	0.93 (0.86;1.13)	0.98 (0.89;1.12)
	Creatinine clearance(ml/min)	47 (27;85)	44.5 (35.5;72.3)
	Plasma renin (mU/ml)	1337.5 (500;3363)	402.0(145.5;1889)
	PAC (pg/mL)	1266 (1043;2141)	992(776.0; 1546.5)
	Paracentesis Induced Circulatory Dysfunction (PICD)(number/total)	6/11 (60%)	4/13 (31%)
	Renal impairment(number/total)	2/11 (20%)	0
Yosry et al. (2018)	Year	2018	
	Study design	RCT	
	Total participants	75	
	Description	T1 (2 days Midodrine) Midodrine 12.5 mg every 8 h for 2 days after LVP. T2(30days midodrine) Midodrine 12.5 mg every 8 h for 30 days after LVP	Regular dose of albumin (8 g for each liter of removed ascitic fluid) immediately after LVP
	Population characteristics		
	Participants	T1=25; T2=25	25
	Male(number/total)	T1=17/25, T2= 18/25	18/25
	Female(number/total)	T1=8/25, T2=7/25	7/25
	Age	T1=51.36±11.68,T2= 50.48±7.93	48.80±10.25
	weight(kg)	T1= 80.04±8.75,T2= 80.16±9.26	79.84±9.06
	Baseline Values		
	Volume of ascites removed(l)	T1=5.80±0.92,T2= 6.13±0.81	5.66±0.83
	Na (mEq/l)	T1=132.68±3.34,T2= 132.24±3.49	130.88±3.06
	Creatine(mg/dL	T1=1.22±0.22,T2= 1.24±0.20	1.24±0.17
	Urinary Na (mEq/L)	T1=26.84±8.68,T2= 23.28±6.23	27.52±11.27
	Outcome on Day 6 (Presented in mean ±SD/ median(IQR)		
	MAP	T1= 82.2±5.06,T2= 78.47±4.22	83.27±4.72
	Serum creatinine	T1=1.35±0.32,T2= 1.24±0.28	1.48±0.32
	Creatinine clearance	T1= 68.73±20.76, T2= 77.03±20.93	61.21±23.06
	on Day 30		
	MAP	T1= 80.87±4.41,T2= 76.45±8.32	80.94±4.35
	Serum creatinine	T1= 1.38±0.42, T2= 1.30±0.53	1.23±0.16
	Creatinine clearance	T1= 70.96±23.49T2= 80.11±29.81	58.14±19.84
	Urinary Volume	T1= 958±217.31,T2= 1169.56±309.96	1104.35±251.32
	U-Na	T1=28±13 ,T2= 29±14	26±15
	30 Day mortality:	T1=0; T2=2	2
Bari et al. (2012)	Year	2012	
	Study design	RCT	
	Total participants	25	
	Description	Saline solution (albumin placebo) Octreotide 20 mg extended release IM every month Midodrine 10mg PO 3 times a day	IV albumin 8 g/L of ascites fluid removed Saline solution 5 mL IM (octreotide placebo) every month Midodrine placebo 3 times a day
	Population characteristics		
	Participants	12	13
	Male (number/total)	12/12	10/13
	Female (number/total)	0	3/13
	AGE median (IQR)	60(51–61)	55(51–65)
	Baseline Values		
	Amount of ascites removed	8 (6–10.5)	6.5 (5–9.5)
	Creatinine level, (mg/dL)	1.1 (1–1.5)	1.1 (0.9–1.5)
	MELD score	14 (13–16)	17 (11–20)
	Serum aldosterone level,(ng/dL )	42 (12–100)	36 (18–89)
	PRA, (ng/mL/hr)	11.8 (7.9–25.1)	19 (17.4–34.5)
	Outcome on Day 6		
	Serum Creatinine	1.2(1.0-1.8)	0.9(0.9-1.4)
	MELD score T	15(12-18)	14(10-16)
	Change in PRA	↑7.1 (-22 to 67)	↓1.3(-51 to 40)
	Change in MAP	↓2(-7 to 5)	↓5(-7 to 2)
	Patients who developed PICD	2/8	2/11
	10 months mortality	5/12	4/13
Hamdy et al. (2014)	Year	2014	
	Study design	RCT	
	Total participants	50	
	Description	Midodrine was administered orally at the dosage of 12.5 mg every 8 hours for 3 days	IV albumin 8 g/L of ascites fluid removed
	Population characteristics		
	Participants	25	25
	Male(number/total)	17/25	21/25
	Female(number/total)	8/25	4/25
	Age mean ±SD	55.88±5.118	58.16±3.436
	Weight (kg)	74.28±5.77	77.92±7.314
	Baseline Values		
	MELD score	15.326±4.34	15.01±3.84
	Ascitic fluid removed(L)	6.84± 0.718	6.96 ±1.040
	Serum albumin (g/dL)	2.372± 0.4297	2.629±0.4572
	MAP (mmHg)	78.99 ± 5.52	81.33 ± 8.05
	Serum creatinine (mg/dL)	0.99 ±0.19	1.10 ± 0.22
	Plasma renin (ng/ml/h)	3.03 ± 0.33	4 ± 0.91
	PAC (pg/mL)	166.72 ± 64.26	204.88 ± 115.9
	Outcome		
	On day 6		
	MAP (mmHg)	71.93 ± 5.8	71.36 ± 7.81
	Serum creatinine(mg/dL)	0.992± 0.1977	1.104± 0.2169
	PRA (ng/ml/h)	4.2 ± 0.76	4.11 ± 0.74
	PAC (pg/mL)	298.64 ± 130.8	177.08 ± 100.5
	Adverse outcomes		
	HRS(number/total)	9/25	0
	Death rate(number/total)	7/25	0
Hanafy et al. (2016)	Year	2016	
	Study design	RCT	
	Total participants	600	
	Description	Midodrine and rifaximin were prescribed as oral midodrine 5 mg every 8 h and rifaximin 550 mg every 12 h, along with the diuretics	Combination of alternative diuretics such as torsemide 20–40 mg/day and amiloride 5–10 mg/day, as long as creatinine clearance was greater than or equal to 50 ml/min.
	Population characteristics		
	Participants	400	200
	Male(number/total)	303/400	150/200
	Female(number/total)	97/400	50/200
	Age mean ±SD	51.5 ± 6.1	52 ± 5
	Baseline Values		
	MAP (mmHg)	75.8 ± 6.2	77 ± 5.5
	Weight(kg)	84.4 ± 8	80.3 ± 4.7
	Creatinine(mg/dL)	1.5 ± 0.2	1.4 ± 0.2
	Creatinine clearance(ml/min)	69.4 ± 11	71.3 ± 14.2
	U-Na(meq/24 h)	16.5 ± 3.6	17.2 ± 2.2
	Urine output (ml/24 h)	528.6 ± 101	580 ± 130
	PRA(ng/ml/h)	4.5 ± 1.2	3.9 ± 0.9
	PAC (ng/dL)	21.6 ± 5.6	19 ± 3.7
	MELD	22.7 ± 2	22.1 ± 2.4
	Outcome		
	2^nd^ Follow up week		
	MAP (mmHg)	84.3 ± 5.6	80.6 ± 5
	Creatinine(mg/dL	1.4 ± 0.16	1.4 ± 0.2
	Creatinine clearance(ml/min)	66.1 ± 10.3	67.4 ± 12.4
	U-Na (meq/24 h)	25.5 ± 4.3	19.5 ± 2.1
	Urine output (ml/24 h)	927 ± 119	787 ± 99
	PRA(ng/ml/h)	3.5 ± 0.7	4.9 ± 1
	PAC (ng/dL)	19.5 ± 4.1	20.3 ± 3.4
	MELD	22.2 ± 1.8	22.7 ± 1.5
	Response Rate		
	Complete Responders(number/total)	320/400	40/200
	Partial Responders(number/total)	56/400	100/200
	Non-Responders(number/total)	24/400	18/200
	Survival (Months)	19.6 ± 3.2	11.6 ± 2.2
	Death Rate(number/total)	12/400	40/200
Kalambokis et al. (2005)	Year	2005	
	Study design		
	Total participants	25	
	Description	Octreotide 300 µg, b.i.d. combined with midodrine hydrochloride 7.5 mg, t.i.d.	subcutaneous octreotide alone
	Population characteristics		
	Participants	13	12
	Male(number/total)	7/13	6/12
	Female(number/total)	6/13	6/12
	Age mean	54(40-77)	56(43-75)
	Baseline Values		
	MAP (mmHg)	79.4 (74-82.6)	79.9(70.4-86.2)
	Cardiac Output (L/min)	6 (5.8-6.2)	6.2 (5.8-6.9)
	Weight(kg)	70.5 (69.5-78)	68 (65-84)
	Serum creatinine(mg/dL)	0.9 (0.7-1)	0.8 (0.7-1)
	U-Na(meq/24 h)	22 (16.5-40.2)	21 (14-48.6)]
	Urine output (ml/24 h)	0.97 (0.79-1.11)	0.86 (0.6-1.05)
	PRA (µU/ml)	109.9 (81.3 -183.8)	66 (22-148.8)
	PAC (ng/dL)	82.5 (40.3-144)	39.4 (15.3-91.9)
	Outcome on day 10		
	MAP (mmHg)	80.6 (70.7-83.3)	82.1 (77.5-94.3)
	Cardiac Output (L/min)	6.8 ( 6.4-7.2)	6 (5.2-6.2)
	Serum creatinine(mg/dL)	0.9 (0.7-1.1)	0.8 (0.7-1.1)
	U-Na(mEq/24 h)	17.1 (11-45.9)	28.7 (18.5-47.3)
	Urine output (ml/min)	0.83 (0.76-0.93)	1.11 (0.76-1.59)
	PRA (µU/ml)	26.8 (17.3 -110.9)	31.8 (6.7-64.8)
	PAC (ng/dL)	19.9 (17.6-100.6)	11.1 (3.1-47.7)
Kalambokis et al. (2007)	Year	2007	
	Study design	RCT	
	Total participants	20	
	Description	Oral midodrine 10 mg, t.i.d. for 7 days	10 mg, t.i.d. Placebo for 7 days
	Population characteristics		
	Participants	12	8
	Male(number/total)	6/12	5/8
	Female(number/total)	6/12	3/8
	Age mean	58 ± 9	57 ± 12
	Baseline Values		
	MAP	84.4 ± 11.9	82.8 ± 10.5
	ClCre	84.4 ± 14.3	89.5 ± 12.9
	Una	29.6 ± 14.8	23.7 ±15
	UV(ml/minute)	0.98± 0.26	0.93 ± 0.41
	PRA	8.55 ± 4.24	8.2 ± 3.98
	PA	398 ± 101	340 ± 83
	Outcome on 7 days		
	MAP	90.2 ± 10	84.1 ± 9.8
	CO	6.1 ± 1.3	6.9 ± 1.2
	ClCre	101 ± 12.6	93.5 ± 11
	Una	48.8 ± 15.9	28.2 ± 16.7
	UV(ml/min)	1.15 ± 0.34	0.9 ± 0.32
	PRA(ng/mL/h)	5.57 ± 3.14	7.81 ± 3.25
	PA (pg/mL)	223 ± 96	318 ± 83
Minakari et al. (2011)	Year	2011	
	Study design	RCT	
	Total participants	34	
	Description	7.5 mg oral midodrine three times daily for 3 days.	50 mg subcutaneous octreotide three times daily for 3 days
	Population characteristics		
	Participants	17	17
	Male(number/total)	12/17	14/17
	Female(number/total)	5/17	3/17
	Age mean	59.47 ± 14.08	49.59 ± 18.03
	Baseline Values		
	PRA (ng/ml/h)	30.99 ± 10.93	28.32 ±8.65
	MAP (mmHg)	73.84 ± 10	78.43 ±8.13
	Weight (Kg)	67.47 ± 11.16	76.58 ± 17.73
	Outcome on day 4, (mean ± SD)		
	PRA	12.94 ± 7.62	20.64 ± 8.23
	MAP	81.57 ± 11.25	85.19 ± 7.9
Misra et al. (2010)	Year	2010	
	Study design	RCT	
	Total participants	15	
	Description	Midodrine 15 mg PO and furosemide 40 mg IV	Placebo (orally given 30 min before) and furosemide 40 mg intravenously
	Population characteristics		
	Participants		
	Male(number/total)	8/15	
	Female(number/total)	7/15	
	Age mean	(52.7±7.6)	
	MELD	(12.1± 2.5)	
	Weight	80.7± 14	
	Systolic blood pressure (mmHg)	114± 15.4	
	Serum albumin (gm ⁄ dL)	3± 0.5	
	Serum creatinine (mg ⁄ dL)	1.06 ±0.2	
	Outcome 0-6 hour		
	Total urine volume (mL)	1770± 262	1962± 170
	Total urinary sodium (mMol)	109± 42	126± 69
Rai et al. (2016)	Year	2016	
	Study design	RCT	
	Total participants	25	
	Description	Oral midodrine 7.5 mg 8 hourly	SMT - restriction of sodium - treatment with diuretics i.e (furosemide 40-160mg/day) and a distal acting diuretic (spironolactone 100-400mg/day) was given with dose escalation by one step at a time permitted for a >10-pound weight gainand -repeated large volume paracentesis (LVP)
	Population characteristics		
	Participants	13	12
	Male(number/total)	8/13	11/12
	Female(number/total)	5/13	1/12
	Weight:	70.0±10.1	66.4±11.4
	Baseline Values		
	MELD score	14.9±2.3	16.1±2.5
	MAP	80.5±4.6	84.5±7.1
	CO	5.85±0.20	5.88±0.33
	Una	70.2±32.2	58.8±22.4
	PRA	11.7±2.5	13.8±2.6
	PA	1530.7±268.9	1555.8±238.4
	Serum Creatinine	0.89±0.28	0.78±0.21
	Urine Output(L/day)	1.08±0.27	1.26±0.35
	Outcome on 1 month		
	MELD Score	14.3±1.48	18.0±2.69
	MAP	T=88.1±6.0	82.1±5.5
	CO	5.81±0.19	5.86±0.29
	U-Na	118.6±33.8	75.8±20.5
	PRA	8.5±1.4	13.8±2.8
	PA	1147.6±316.7	1527.5±300.2
	Serum Creatinine	0.84±0.19	0.87±0.34
	Urine Output(L/day)	1.44±0.27	1.20±0.23
	At 3 months		
	MELD Sore	14.6±1.06	15.8±2.91
	MAP	90.3±3.6	83.7±7.6
	CO	5.73±0.22	5.78±0.33
	U-Na	111.2±26.9	79.9±10.5
	Serum Creatinine	0.84±0.19	0.80±0.10
	Urine Output	1.45±0.24	1.12±0.29
	Mortality rate and Morbidity rate		
	Death :(number/total)	1/13	1/12
	Encephalopathy(number/total)	0	1/12
	Renal failure(number/total)	0	4/12
	SBP(number/total)	0	2/12
	Sepsis(number/total)	1 /13	2 /12
Singh et al. (2008)	Year	2008	
	Study design	RCT	
	Total participants	40	
	Description	Midodrine 5–10 mg three times a day	Albumin 8 g/L of ascitic fluid was removed (mean 48.4 ± 12.1 g)
	Population characteristics		
	Participants	20	20
	Male(number/total)	18/20	17/20
	Female(number/total)	2/20	3/20
	AGE mean ±SD	48.15 ± 11.26	45.05 ± 14.16
	Baseline Values		
	MAP	86.10 ± 6.90	85.85 ± 6.63
	U Na	9.60±12.42	18.80 ± 29.75
	PRA	44.44 ± 8.44	43.18 ± 10.73
	PA	1,640.00±539.40	1,890.00±590.18
	SerumCreatinine	0.79±0.17	0.85±0.17
	UrineOutput(ml/day)	1,495.00 ± 337.91	1,540.00 ± 440.57
	Outcome Day 6		
	MAP	87.20 ± 7.36	87.00 ± 7.23
	UNa	25.00 ± 23.38	22.55 ± 28.65
	PRA	41.39 ± 10.21	45.90 ± 8.59
	PA	1,700.00 ± 493.11	1,965.00 ± 497.65
	Serum Creatinine	0.86 ± 0.21	0.98 ± 0.25
	Urine Output (ml/day)	1,640.00 ± 388.52	1,555.00 ± 527.63
	Output (ml/day)	1,640.00 ± 388.52	1,555.00 ± 527.63
	PICD(number/total)	0	2/20
	Death(number/total)	1/20	0
	Response rate		
	Repeat paracentesis (within 3 month of treatment)(number/total)	1/20	2/20
Singh et al. (2012)	Year	2012	
	Study design	RCT	
	Total participants	40	
	Description	Midodrine Subjects randomized to midodrine were given oral midodrine 7.5 mg 8 hourly	SMT - restriction of sodi-um - treatment with diuretics i.e (furosemide 40-160mg/day) and a distal acting diuretic (spironolactone 100-400mg/day) was given with dose escalation by one step at a time permitted for a >10-pound weight gainand -repeated large volume paracentesis (LVP)
	Population characteristics		
	Participants	20	20
	Male(number/total)	17/20	20/20
	Female(number/total)	3/20	0
	AGE mean ±SD	45.6 ± 10.049	47.6 ± 11.033
	Baseline Values		
	Recurrent ascites(number/total)	14/20	14/20
	Refractory ascites(number/total)	6/20	6/20
	MELD score	12.9 ± 3.13	14.85 ± 4.68
	Weight (kg)	68.45 ± 18.70	64.43 ± 12.15
	Mean arterial pressure (mmHg)	85.6 ± 10.7	83.59 ± 11.44
	CO	5.68± 1.66	5.81± 1.82
	Serum Sodium	134.6± 10.57	134.15± 5.5
	Una	73.14± 35.63	70.47± 30.24
	PRA	13.73± 4.41	13.12± 3.88
	PA	1601.5± 789.7	1545.3± 630.9
	Serum Creatinine	0.85± 0.272	1.03± 0.310
	Serum Creatinine	0.85± 0.272	1.03± 0.310
	Urine Output	1235± 665.12	1381.2± 636.8
	Outcome on 1 month		
	Weight:	67.15± 19.78	65.5± 10.79
	MELD Score	13.9± 4.1	16.1± 5.6
	MAP	92.88± 7.91	83.01± 8.50
	Una	93.21± 32.19	68.75± 18.93
	PRA	9.66± 2.51	14.75± 3.48
	PA	921.5± 547.8	1440.59± 497.3
	Serum Creatinine	0.84± 0.205	1.01± 0.227
	Urine Output(ml/day)	1830± 564.84	1496.8± 549.6
	Response Rate		
	At 1 month		
	No of Patients	18	17
	Complete(number/total)	2/18	0
	Partial		
	None(number/total)	1/18	3/17
	At 3 months		
	No of Patients	16	16
	Complete(number/total)	5/16	1/16
	Partial(number/total)	10/16	7/16
	None(number/total)	1/16	8/16
	At 6 months		
	No of Patients	12	5
	Complete(number/total)	5/12	1/5
	Partial(number/total)	4/12	4/5
	None(number/total)	0	0
	Mortality		
	1-month(number/total)	3/20	4/20
	3 months(number/total)	7/20	11/20
	6 months(number/total)	8/20	15/20
Singh et al. (2013)	Year	2013	
	Study design	RCT	
	Total participants	30	
	Description	Oral midodrine 7.5 mg 8 hourly	SMT - restriction of sodi-um - treatment with diuretics i.e (furosemide 40-160mg/day) and a distal acting diuretic (spironolactone 100-400mg/day) was given with dose escalation by one step at a time permitted for a >10-pound weight gainand -repeated large volume paracentesis (LVP)
	Population characteristics		
	Participants	15	15
	Male(number/total)	14/15	15/15
	Female(number/total)	1/15	0
	Baseline Values		
	Recurrent ascites(number/total)	6/15	6/15
	Refractory ascites(number/total)	9/15	9/15
	Weight	67.06 ± 12.82	73.86 ± 7.94
	MELD Score	13.56 ± 5.71	13.92 ± 4.18
	MAP	85.3 ±8.72	92.6 ±6.06
	CO	6.67 ±1.21	6.70± 1.36
	Una	42.2 ±12.6	35.6 ±14.3
	Serum Creatinine	1.03 ±0.30	1.11 ±0.20
	Urine Output(ml/day)	995.3±226.7	947.3±250.6
	PRA	12.0 ±3.00	13.6± 2.75
	PA	1512.0 ±444.1	1528.0± 497.1
	Outcome on 1 months		
	MELD Score	12.4 ±3.67	13.5 ±3.99
	MAP	94.7±4.48	87.6±5.24
	U-Na	72.5±18.1	45.2±19.6
	Serum Creatinine	1.01 ±0.25	1.13± 0.22
	Urine Output	1267.8 ±333.1	1107.8± 316.3
	PRA	9.22 ±2.74	13.8 ±2.86
	PA	820.7 ±223.9	1410.8± 332.2
	1 month mortality(number/total)	1/15	1/15
	Response Rate:		
	1 months		
	Total patients	14	12
	Complete(number/total)	-	-
	Partial(number/total)	11/14	5/12
	None(number/total)	-	-
Solà et al. (2018)	Year	2018	
	Study design	RCT	
	Total participants	173	
	Description	Midodrine 15mg/day or 30mg/day based on MAP goal Albumin i.v. at a dose of 40g every 15 days.	Placebo of midodrine; 0.9% saline as a placebo of albumin
	Population characteristics		
	Participants	87	86
	Male(number/total)	66/87	71/86
	Female(number/total)	21/87	15/86
	Baseline Values		
	MELD score	17±6.0	16±6.2
	MAP	80±10mmHg	81±10mmHg
	Serum creatinine (mg/dL)	0.96±0.3	1.0±0.4
	MAP (mmHg)	80±10	81±10
	Outcome		
	At Week 4, MELD score	13±4	13±4
	At Week 12, MELD score	13±3	13±4
	At Week 24, MELD score	13±2	12±4
	Patients with adverse event(number/total)	83/87	84/86
	Renal impairment	12/83	11/84
	Hyponatremia	11/83	14/84
	Hepatic encephalopathy	24/83	21/84
	Sepsis	12/83	13/84
	Gastrointestinal bleeding	8/83	4/84
	Mortality at 2 month	38/87	31/86
	Mortality at 6 month	68/87	51/86

Quantitative Analysis

Fourteen studies [1,3,4,6,8,14-22] comprising a total of 1199 patients were included in our quantitative analysis.

Mean Arterial Pressure (MAP)

**Figure 3:**
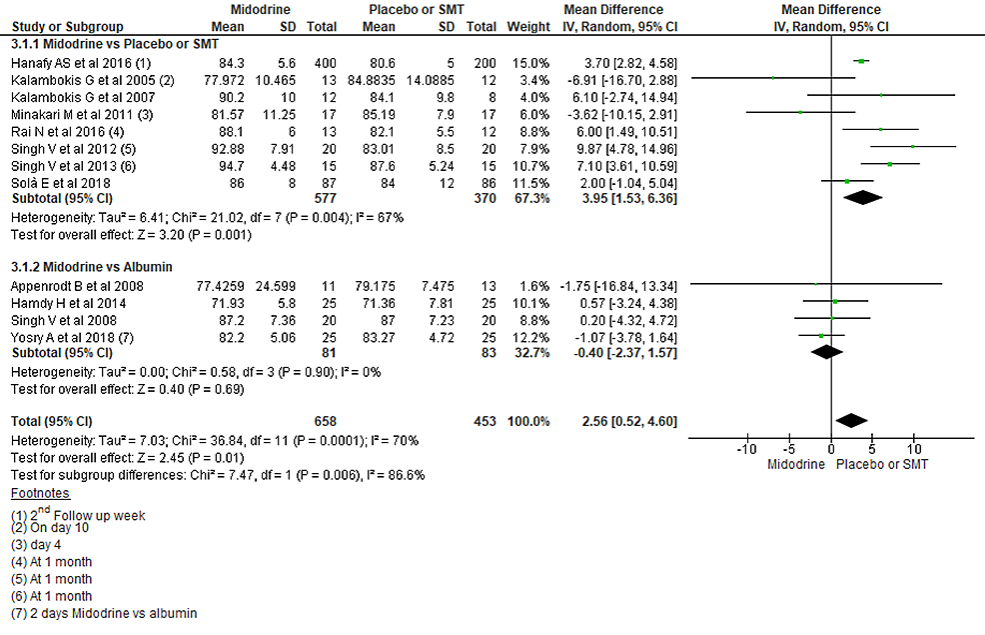
Forest plots comparing MAP between midodrine and Placebo/SMT, and midodrine and albumin The square box across the horizontal lines represents the mean difference (MD) value for the individual study, the horizontal line represents 95% conﬁdence interval (CI), and the diamond represents the pooled MD with its CI. MAP= Mean arterial pressure, SMT= Standard medical treatment Included studies are reference nos. [1,3,4,6,8,16-22]

A total of twelve studies reported MAP outcomes, mostly around one week of treatment. The addition of midodrine to standard medical treatment (SMT) showed a mean MAP of 2.56 mmHg higher in the midodrine group (MD, 3.95 mmHg; 95% CI, 1.53- 6.36; p=0.001) compared to SMT. Midodrine when compared to albumin did not reach significant differences level in terms of MAP (MD -0.40, 95% CI -2.37 to 1.57; n= 164; I2 = 0%) (Figure 3).

MELD Score

**Figure 4:**
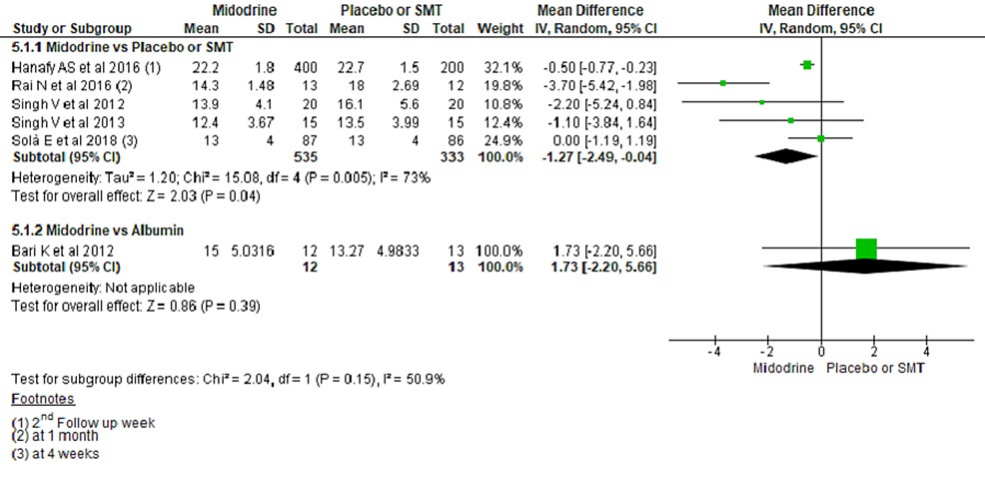
Forest plot comparing mean MELD score between midodrine and placebo/SMT. (Only one study compared midodrine with albumin for MELD score) The square box across the horizontal lines represents the mean difference (MD) value for the individual study, the horizontal line represents 95% conﬁdence interval (CI), and the diamond represents the pooled MD with its CI. MELD= Model for End-Stage Liver Disease, SMT= Standard medical treatment Included studies are reference nos. [6,15,19-22].

Six studies reported MELD (Model for End-Stage Liver Disease) scores among 14 studies included. The use of midodrine showed a significant reduction in MELD score among ascitic patients compared with SMT. Comparing midodrine with SMT showed an average of 1.27 points lower MELD score in midodrine group (MD -1.27, 95% CI -2.49 to -0.04; n= 868; I2 = 73%) (Figure 4).

Plasma Renin Activity (PRA) (ng/ml/hr)

**Figure 5:**
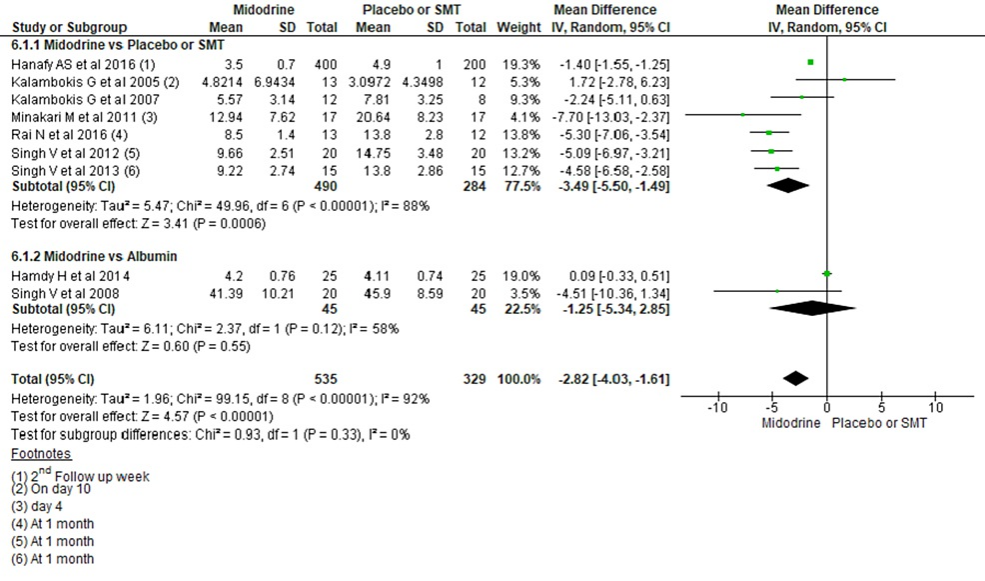
Forest plots comparing mean PRA between midodrine and placebo/SMT, and midodrine and albumin A square box across the horizontal lines represents the mean difference (MD) value for the individual study, the horizontal line represents 95% conﬁdence interval (CI), and the diamond represents the pooled MD with its CI. PRA= Plasma renin activity, SMT= Standard medical treatment Included studies are [3,6,8,16-21].

Overall, midodrine use caused an average of 3.49 ng/ml/hr lower PRA in the treatment group than SMT/Placebo (MD -3.49, 95% CI -5.50 to -1.49; P=0.0006). At the same time, PRA activity was not different when midodrine was compared to albumin (MD -1.25, 95% CI -5.34 to 2.85; n= 90; I2 = 58%) (Figure 5).

Plasma Aldosterone Concentration (PAC) (pg/ml)

**Figure 6:**
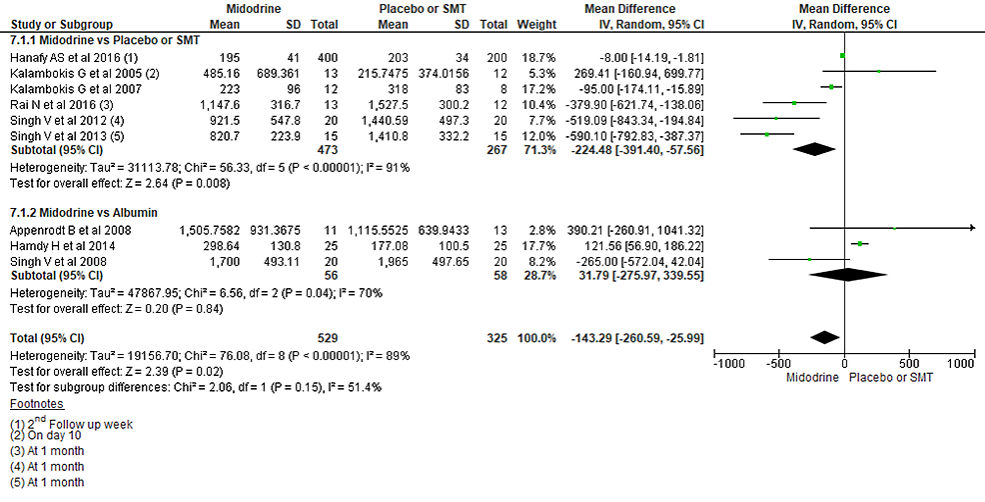
Forest plot comparing mean PAC among midodrine and other treatments in case of ascites due to cirrhosis The square box across the horizontal lines represents the mean difference (MD) value for the individual study, the horizontal line represents 95% conﬁdence interval (CI), and the diamond represents the pooled MD with its CI. PAC= Plasma aldosterone concentration Included studies are [1,3,6,8,16,17,19-21].

Overall, midodrine use averages 223.48 pg/ml lower PAC in the treatment group than SMT (MD -224.48, 95% CI -391.40 to -57.56; P=0.008). Comparing midodrine to albumin did not show significant differences (MD 31.79, 95% CI -275.97 to 339.55P=0.84) (Figure 6).

Short-Term Mortality

**Figure 7:**
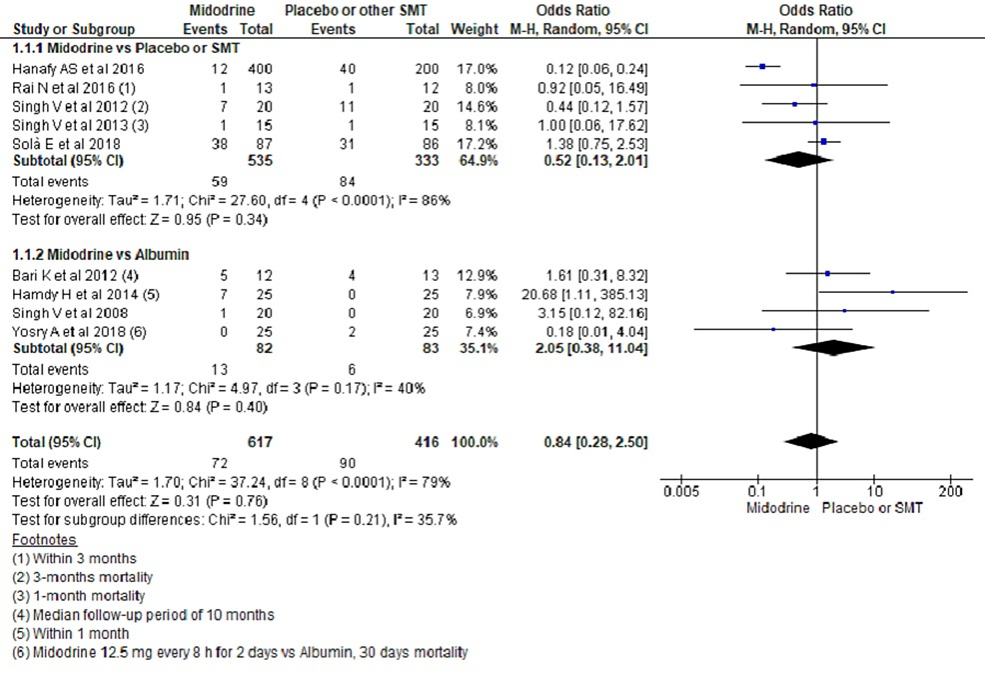
Forest plots showing mortality comparing midodrine to SMT/Placebo and albumin The square box across the horizontal lines represents the Odds Ratio (OR) value for the individual study, the horizontal line represents 95% conﬁdence interval (CI), and the diamond represents the pooled OR with its CI. SMT= Standard medical treatment Included studies are  [3,4,6,15-17,19-21].

A total of eight studies reported mortality outcomes. There were no significant differences in short-term mortality (within three months, though it was reported heterogeneously across studies noted in footnotes) when midodrine use was compared to SMT/placebo or albumin (OR, 0.52; 95% CI, 0.13 to 2.01; P=0.34 and OR, 2.05; 95% CI, 0.38 to 11.04; P=0.40 respectively) (Figure 7).

Serum Creatinine

**Figure 8:**
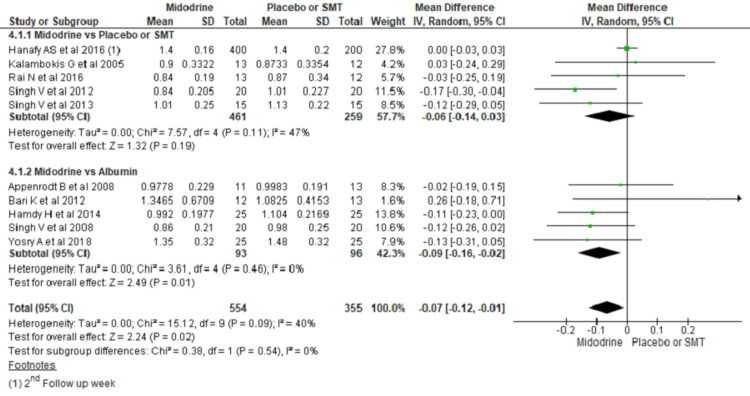
Forest plots comparing mean serum creatinine between midodrine and placebo/SMT, and midodrine and albumin The square box across the horizontal lines represents the mean difference (MD) value for the individual study, the horizontal line represents 95% conﬁdence interval (CI), and the diamond represents the pooled MD with its CI. SMT= Standard medical treatment Included studies are  [1,3,4,6,8,15,16,19-21].

A total of ten studies reported serum creatinine value during the study period, mostly around one week of treatment. Midodrine use was not statistically significant in lowering serum creatinine compared to SMT/placebo; however, it was nearing statistical significance (MD, -0.06; 95% CI, -0.14 to 0.03; P=0.19). On the contrary, midodrine use leads to a statistically significant reduction in serum creatinine compared to albumin (MD, -0.09; 95% CI, -0.16 to -0.02; P=0.01) (Figure 8).

Paracentesis Induced Circulatory Dysfunction (PICD)

**Figure 9:**
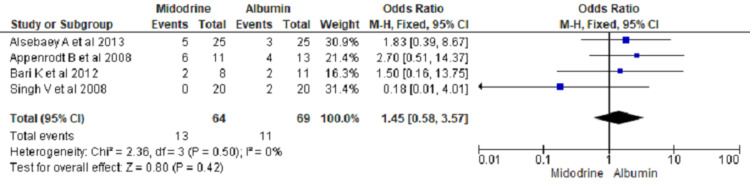
Forest plot showing PICD comparing midodrine with other treatments in case of ascites due to cirrhosis The square box across the horizontal lines represents the Odds Ratio (OR) value for the individual study, the horizontal line represents 95% conﬁdence interval (CI), and the diamond represents the pooled OR with its CI. PICD= Paracentesis induced circulatory dysfunction Included studies are [1,3,14,15].

Paracentesis Induced Circulatory Dysfunction (PICD) as an outcome was reported in four RCTs. Midodrine use did not show significant difference in PICD outcome compared to SMT (OR 1.45, 95% CI 0.58 to 3.57; n= 133; I2 = 0%) (Figure 9).

Publication Bias

**Figure 10:**
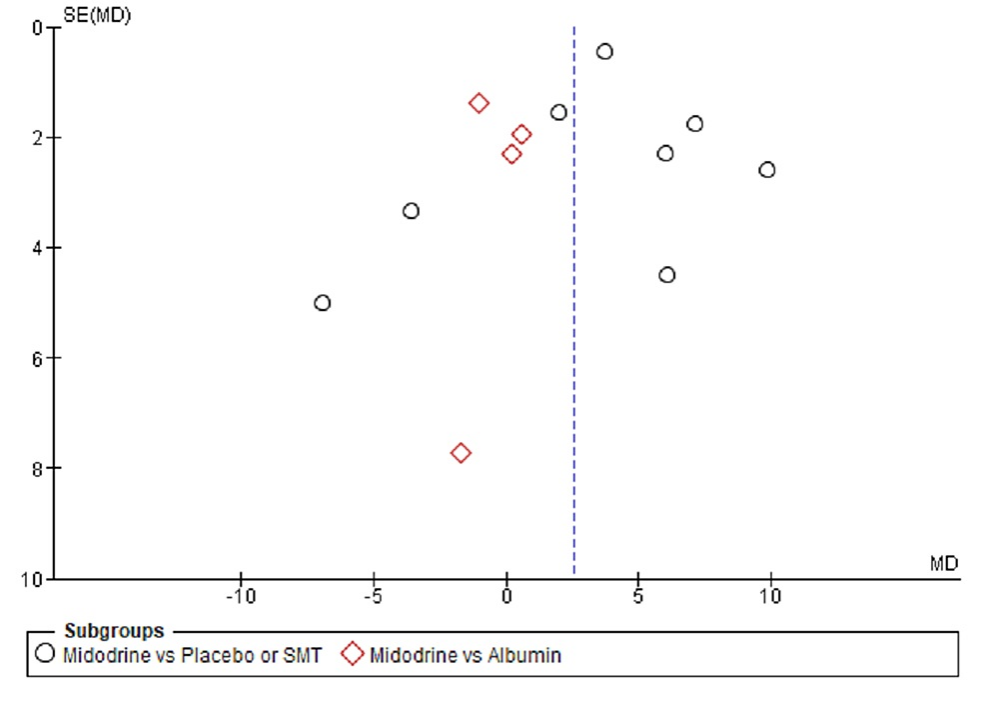
Funnel plot showing the asymmetric distribution of studies suggesting publication bias for MAP outcome MAP= Mean arterial pressure, SMT= Standard medical treatment

**Figure 11:**
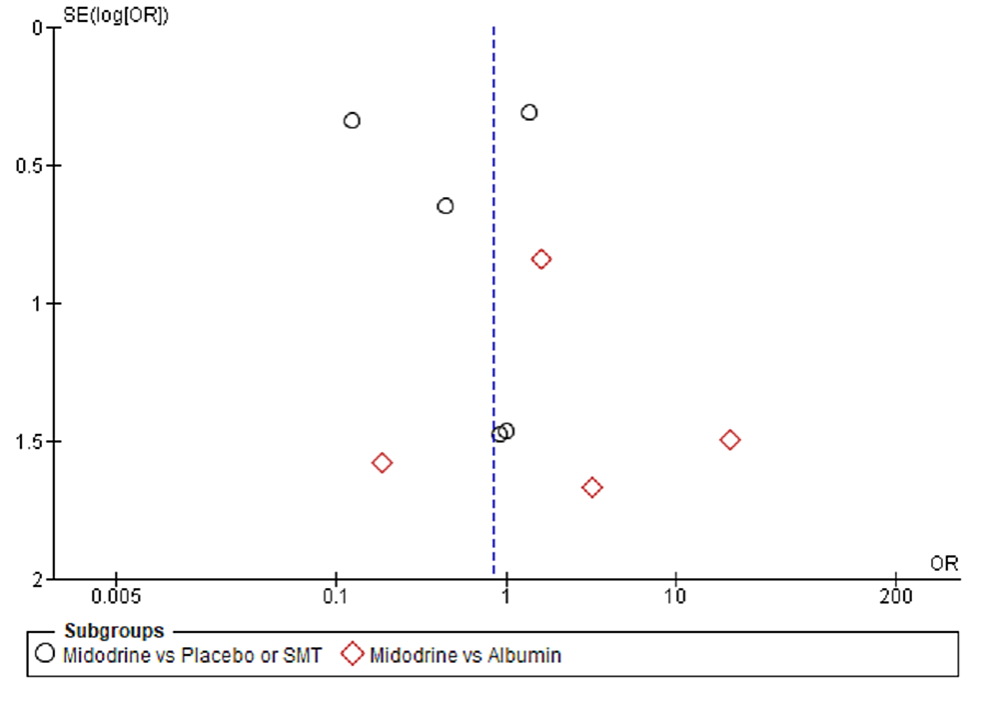
Funnel plot showing the asymmetric distribution of studies suggesting publication bias for short-term mortality outcome SMT= Standard medical treatment

Publication bias of the included studies was assessed and presented in Funnel plots. Significant publication bias was present as suggested by an asymmetry of the plot for outcomes evaluated (Figures 10-11).

Discussion

Cirrhotic ascites is usually associated with hypotension due to vasodilation mediated by low effective circulatory volume. Diuretics in such cases can further worsen renal perfusion and decrease renal sodium excretion. Midodrine is an oral vasopressor that blocks vasodilation and increases blood pressure, potentially leading to improved renal perfusion and decreased ascites [20,21,23]. This possibly leads to mortality and morbidity benefits. In this meta-analysis, we focused on the role of midodrine in combination with drugs like rifaximin, octreotide, and clonidine in cirrhotic ascites. Different studies included rifaximin, octreotide, clonidine, albumin, terlipressin, hydroxyethyl starch (HES), a combination of alternative diuretics like torsemide, amiloride, furosemide, and spironolactone, repeated large-volume paracentesis as standard medical treatment (SMT). As expected, we found significant improvement in blood pressure in patients receiving midodrine compared to standard medical treatment as a potential effect of alpha-1 mediated vasoconstriction. Midodrine use was statistically significant in lowering serum creatinine compared to albumin, however, reduction in creatinine did not reach the level of significance while compared with SMT/placebo. This is likely due to the effect of midodrine, which has been found to improve renal hemodynamics, and glomerular filtration rate (GFR) and promote sodium excretion in patients with cirrhosis [5,18,20]. In our analysis, we found midodrine to decrease plasma renin and aldosterone concentration compared to standard medical treatment alone. This is significant because this explains the beneficial effect of midodrine in paracentesis-induced circulatory dysfunction and the apparent lack of difference observed between patients treated with albumin and midodrine regarding the occurrence of PICD. Midodrine was found to improve urine output and cause weight loss in multiple studies [8,17,20]. However, the patients in these studies received concomitant diuretic therapy, which also leads to these changes, and the benefit cannot be solely credited to midodrine. We also found a significant reduction in MELD scores comparing patients treated with midodrine to standard medical treatment. A reduction in MELD scores is a possible prognostic factor for patients with cirrhosis and ascites. However, a previous study suggested reversible deterioration of MELD score with midodrine, octreotide, and albumin treatment for one month in refractory ascites [24]. This might be due to the co-administration of octreotide and midodrine for one month. Our analysis of MELD scores included studies in which patients received midodrine alone and for prolonged periods.

Our analysis found no difference in PICD between patients receiving albumin and midodrine while analyzing the results of four trials that reported on PICD [1,3,14,15]. PICD was defined as increased plasma renin by 50% from baseline at day six in studies [1,14]. Therapeutic paracentesis leads to depletion of intracellular volume, thereby activating the renin-angiotensin-aldosterone system and increasing renin levels. Expansion of plasma volume with albumin decreases the risks of paracentesis-induced circulatory dysfunction in various studies. However, we found no difference in PICD between patients treated with albumin and midodrine [1,16]. This finding was similar to the previous meta-analysis done by Guo et al. [9]. However, we did not find a significant difference in short-term mortality between midodrine and SMT, midodrine and albumin. Our findings are similar to the previous meta-analysis done by Guo et al., who found no improvement in mortality at one month [9]. Sola et al. reported renal impairment, hepatic encephalopathy, gastrointestinal bleeding, hyponatremia, and sepsis as some of the adverse effects of midodrine compared to placebo [22].

Our meta-analysis is the most comprehensive meta-analysis to date, including a total of 14 studies, and the second meta-analysis to evaluate the effect of midodrine in cirrhotic ascites. We have compared multiple outcomes regarding the use of midodrine in cirrhotic ascites to albumin and standard medical treatment. Terlipressin and albumin are treatments for refractory ascites, but both require intravenous access and are expensive. Our findings of midodrine being non-inferior to albumin regarding the occurrence of PICD and decrement in plasma renin and aldosterone are significant because midodrine is available in cheap oral formulation making it much easier to use.

Our study has several limitations. The endpoints for assessment of our outcomes were variable ranging from day four, day 10, one month to three months [8,18-21]. In some of the studies, patients received concomitant adjuvant treatment like octreotide [8,15], and rifaximin [6]. Another significant limitation was the wide variation in the dosage and duration of midodrine ranging from three days to months, which caused heterogeneity in the reported results. Finally, there were inherent limitations in included studies like small sample size, lack of proper randomization, short duration of midodrine treatment, etc.

## Conclusions

Midodrine alone leads to statistically significant improvement in various clinical parameters in patients with cirrhotic ascites compared to standard medical care. At the same time, it appears to be non-inferior to albumin. We report that the addition of midodrine to SMT for diuretic-resistant cirrhotic ascites would be beneficial. The results from our study call for further well-designed studies evaluating the combination of midodrine and albumin for optimal clinical benefits.

## Appendices

Supplementary file 1. Electronic database search details

PubMed

Hits: 44

Search: "midodrine" AND "ascites" AND "cirrhosis"

Link: https://pubmed.ncbi.nlm.nih.gov/?term=%22midodrine%22+AND+%22ascites%22+AND+%22cirrhosis%22

PubMed Central

Hits: 255

Search: "midodrine" AND "ascites" AND "cirrhosis"

Link: https://www.ncbi.nlm.nih.gov/pmc/?term=%22midodrine%22+AND+%22ascites%22+AND+%22cirrhosis%22

Embase

Hits: 323

Search: ('midodrine'/exp OR 'midodrine') AND ('ascites'/exp OR 'ascites')

Link: https://www.embase.com/#advancedSearch/resultspage/history.15/page.1/25.items/orderby.date/source.

Scopus

Hits: 242

Search: "midodrine" AND "ascites" AND "cirrhosis"

Link: https://www.scopus.com/results/results.uri?numberOfFields=0&src=s&clickedLink=&edit=&editSaveSearch=&origin=searchbasic&authorTab=&affiliationTab=&advancedTab=&scint=1&menu=search&tablin=&searchterm1=%22midodrine%22+AND+%22ascites%22+AND+%22cirrhosis%22&field1=TITLE_ABS_KEY&dateType=Publication_Date_Type&yearFrom=Before+1960&yearTo=Present&loadDate=7&documenttype=All&accessTypes=All&resetFormLink=&st1=%22midodrine%22+AND+%22ascites%22+AND+%22cirrhosis%22&st2=&sot=b&sdt=b&sl=56&s=TITLE-ABS-KEY%28%22midodrine%22+AND+%22ascites%22+AND+%22cirrhosis%22%29&sid=b0edf479bae139c7f8ea1d1d484d75bf&searchId=b0edf479bae139c7f8ea1d1d484d75bf&txGid=87a4a3b838c2a1b7cc17e330c7248561&sort=plf-f&originationType=b&rr=

**Table 2:** Supplementary file, basic details of included studies Alsebaey et al. (2013) [14]; Appenrodt et al. (2008) [1]; Yosry et al. (2018) [4]; Bari et al. (2012) [15]; Hamdy et al. (2014) [16]; Hanafy et al. (2016) [6]; Kalambokis et al. (2005) [8]; Kalambokis et al. (2007) [17]; Minakari et al. (2011) [18]; Misra et al. (2010) [7]; Rai et al. (2016) [19]; Singh et al. (2008) [3]; Singh et al. (2012) [20]; Singh et al. (2013) [21]; Solà et al. (2018) [22]

Study ID	Title	Country	Design	Start date	End date	Inclusion criteria	Exclusion criteria	Limitations
Alsebaey et al. 2013	Prevention of paracentesis-induced circulatory dysfunction: could we use other albumin alternatives?	Egypt	RCT	2013	-	The presence of tense ascites determined by clinical examination and abdominal ultrasound, requiring frequent therapeutic paracentesis, age younger than 70 years and older than 18 years, and absence of arterial hypertension, history of coronary disease, cardiac failure, respiratory disease, hepatic encephalopathy, sepsis, spontaneous bacterial peritonitis (defined by polymorphonuclear cell count >250/ml in ascites), elevated creatinine concentration higher than 1.5 mg/dl, and gastrointestinal bleeding within 7 days before the study	Not Specified	Not Specified
Appenrodt et al. 2008	Prevention of paracentesis-induced circulatory dysfunction: midodrine vs albumin.	Germany	RCT	October 2004	May 2006	The presence of liver cirrhosis with tense ascites (>5 L), determined by abdominal ultrasound and clinical examination, requiring therapeutic paracentesis.	Patients with a prothrombin time of <30%, platelet count of < 30 000/L, a serum creatinine concentration of >1.5 mg/dl those younger than 18 years and older than 70 years, Recent onset or change in diuretic therapy, and use of albumin or paracentesis as well as gastrointestinal bleeding and sepsis The diagnosis of spontaneous bacterial peritonitis (defined by polymorphonuclear cell count > 250/ml in ascites) within 7 days before the study onset.	Small sample size, the dose, and duration of drug administration were fixed with no adaptation by hemodynamic parameters.
Yosry et al. 2018	Oral midodrine is comparable to albumin infusion in cirrhotic patients with refractory ascites undergoing large-volume paracentesis: results of a pilot study	Egypt	RCT	July 2015	April 2016	1. Cirrhotic patients with refractory or recurrent ascites. 2. Patients younger than 70 years of age and older than 18 years of age. 3. Absence of sepsis. 4. Prothrombin concentration of more than 30% and platelet count of more than 25 000/l. 5. Serum creatinine less than 1.5 mg/dl.	1. Diuretics dose change within 7 days before the study. 2. Spontaneous bacterial peritonitis and/or gastrointestinal bleeding within 7 days before the study. 3. Marked respiratory distress necessitating tapping to be performed on the same day of presentation. 4. Hepatic encephalopathy or malignancy. 5. Uncontrolled diabetes (HbA1c>8.5%), arterial hypertension, history of coronary heart disease, or cardiac failure. 6. Refusal to participate in the study	Not specified.
Bari et al. 2012	The Combination of Octreotide and Midodrine Is Not Superior to Albumin in Preventing Recurrence of Ascites After Large-Volume Paracentesis	USA	RCT	October 2003	June 2010	Age: 18-80 years Cirrhosis of any etiology, refractory ascites	"Patients with hepatic hydrothorax Small amount of ascites Recent (within 1 mo) gastrointestinal hemorrhage Active bacterial infection Cardiac failure Findings suggestive of organic renal disease Hepatocellular carcinoma, Baseline serum creatinine level greater than 3.0"	Small sample size, the dose, and duration of drug administration were fixed with no adaptation by hemodynamic parameters.
Hamdy et al. 2014	Comparison of Midodrine and Albumin in the Prevention of Paracentesis-induced Circulatory Dysfunction in Cirrhotic Patients	Egypt	RCT	November 2010	March 2012	"1. Patients with refractory ascites, less than 70 years of age and more than 18 years of age with cirrhosis and tense ascites (>5 L), determined by abdominal ultrasound and clinical examination, requiring therapeutic paracentesis. "	"1. The presence of arterial hypotension or hypertension, a history of coronary heart disease, cardiac failure, respiratory disease, renal disease, urinary retention, pheochromocytoma, thyrotoxicosis, or diabetes mellitus; 2. The presence of sepsis, spontaneous bacterial peritonitis, hepatic encephalopathy, and gastrointestinal bleeding within 7 days before the study; 3. Recent use of diuretics or change in diuretic therapy, b-blockers, plasma expanders, or paracentesis."	Small sample size, the dose, and duration of drug administration were fixed with no adaptation by hemodynamic parameters.
Hanafy et al. 2016	Rifaximin and midodrine improve clinical outcomes in refractory ascites including renal function, weight loss, and short-term survival	Egypt	RCT	November 2011	May 2015	"1. Age 18–70 years, evidence of end-stage liver disease and ascites that is refractory to conventional therapy at the maximum tolerated dose of spironolactone and furosemide, rapidly recurrent ascites, and systolic blood pressure (SBP) less than 100 mmHg."	"1. Non-cirrhotic causes of ascites, primary renal medical diseases, any grade of unresolved hepatic encephalopathy until it has improved and stabilized, 2. active gastrointestinal bleeding, HRS, previous antibiotic prophylaxis for spontaneous bacterial peritonitis, 3. the presence of hepatocellular carcinoma or portal vein thrombosis, active cardiovascular disease, 4. Systemic hypertension, drugs that affect systemic and renal hemodynamics, and active alcohol consumption."	Randomization was not centralized
Kalambokis et al. 2005	The Effects of Chronic Treatment with Octreotide versus Octreotide plus Midodrine on Systemic Hemodynamics and Renal Hemodynamics and Function in Nonazotemic Cirrhotic Patients with Ascites	Greece	RCT	January 2003	January 2004	"1. Absence of gastrointestinal bleeding, hepatic encephalopathy, or infection within the 2 week preceding the study or during the study, 2. absence of refractory ascites or HRS, according to the criteria recently proposed (27), 3. no treatment with diuretics or other drugs with known effects on systemic and renal hemodynamics and/or on renal function within the 5 days before the inclusion, 4. positive sodium balance after at least 5 days of restricted sodium intake (80 mEq/day), 5. absence of diabetes, organic nephropathy, cardiopathy, arterial hypertension 6. absence of hepatocellular carcinoma or portal vein thrombosis 7. Willingness to participate."	-	Not specified.
Kalambokis et al. 2007	Effects of a 7-day treatment with midodrine in non-azotemic cirrhotic patients with and without ascites	Greece	RCT	2006	2006	"1. Absence of gas- trointestinal bleeding, hepatic encephalopathy or infection within the 1 month preceding the study or during the study, 2. absence of mas- sive or tense ascites, refractory ascites or HRS according to the proposed criteria , 3. no treatment with diuretics or other drugs with known effects on systemic and renal haemodynamics and/or renal function within the 7 days before the inclusion, 4. absence of diabetes, intrinsic renal or cardiovascular disease or arterial hypertension on history and physical examination, abnormal urinalysis, chest radio- graph, or electrocardiograph 5. Absence of hepatocellular carcinoma or portal vein thrombosis."	-	Not Specified
Minakari et al. 2011	Comparison of the effect of midodrine versus octreotide on hemodynamic status in cirrhotic patients with ascites	Iran	RCT	January 2007	January 2009	"Age more than 15 years old Do not had GI bleeding during last 7 days and/or had an unstable hemodynamics Do not have hepatic encephalopathy Have no infection (sepsis, spontaneous bacterial peritonitis) within the last 30 days Do not have diabetes mellitus Do not have cardiovascular diseases and hypertension Have no proven hepatocellular carcinoma Do not have hepatorenal syndrome Have no known allergy to drugs."	Having hepatic encephalopathy Hepatorenal syndrome, hemodynamic instability Infection or gastrointestinal bleeding during the course of admission	1. The study could not measure some variables such as renal blood flow, cardiac output systemic vascular resistance and urinary sodium excretion. 2. Other limitations of this study were small size of the groups and short duration of treatment
Misra et al. 2010	The effects of midodrine on the natriuretic response to furosemide in cirrhotics with ascites	USA	RCT	17 April 2002	Not mentioned * published in 2010	16 Adult cirrhotic patients with clinically detectable ascites (age ‡18, Child– Pugh score ‡7) were screened prior to enrolment, but one subject chose not to participate further during the initial equilibration phase, hence the total number of participants were 15	Congestive heart failure Creatinine clearance <60 mL⁄min Untreated endocrinopathies Actively consuming alcohol, Who have a TIPS.	
Rai et al. 2016	Midodrine and tolvaptan in patients with cirrhosis and refractory or recurrent ascites :a randomized pilot study	India	RCT	2016	2016	"Patients with cirrhosis and refractory or recurrent ascites with stable renal function (creatinine level <1.5 mg/dL for at least 7 days)"	"Gastrointestinal bleeding, hepatorenal syndrome, grade 2 or higher hepatic encephalopathy, infection within 1 month preceding or during the study, presence of diabetes, intrinsic renal or cardiovascular disease or arterial hypertension on history and physical examination, abnormal urine analysis, abnormal chest radiograph or electrocardiogram, presence of hepatocellular carcinoma or portal vein thrombosis, treatment with drugs with known effects on systemic and renal hemodynamics (beta-blockers were withdrawn and low dose diuretics were continued as tolerated, provided the serum creatinine was < 1.5 mg/dL) within 7 days before inclusion and active alcoholis"	-The small sample size of this pilot trial raises the potential for Type 2 error and limits the interpretation of the results. -this is an open-label study. -there is a mismatch in the number of patients in refractory/recurrent ascites.
Singh et al. 2008	Midodrine Versus Albumin in the Prevention of Paracentesis-Induced Circulatory Dysfunction in Cirrhotics: A Randomized Pilot Study	India	RCT	2005	2006	"Presence of tense ascites requiring frequent therapeutic paracentesis - patients less than 70 yr of age; -absence of arterial hypertension, a history of coronary heart disease, cardiac failure, symptomatic arteritis, respiratory or renal disease, diabetes mellitus, hepatocellular carcinoma, and hepatic encephalopathy - absence of sepsis and gastrointestinal bleeding within 7 days before the study - absence of recent use of diuretics, beta-blockers, plasma expanders, or paracentesis"	-	Not specified
Singh et al. 2012	Midodrine in patients with cirrhosis and refractory or recurrent ascites: A randomized pilot study	India	RCT	2007	2009	"Presence of refractory or recurrent ascites - patients less than 70 years of age -absence of gastrointestinal bleeding, hepatorenal syndrome, hepatic encephalopathy of grade 2 or higher or infection within 1 month preceding the study or during the study - presence of diabetes, intrinsic renal or cardiovascular disease or arterial hypertension on history and physical examination, abnormal urine analysis, chest radiograph, or electrocardiogram, - presence of hepatocellular carcinoma or portal vein thrombosis - no treatment with drugs with known effects on systemic and renal hemodynamics within 7 days before inclusion."	-	Not specified
Singh et al. 2013	Midodrine and Clonidine in Patients With Cirrhosis and Refractory or Recurrent Ascites: A Randomized Pilot Study	India	RCT	2010	2011	"-Presence of refractory or recurrent ascites - patients less than 70 years of age; - absence of gastrointestinal bleeding, hepatorenal syndrome, hepatic encephalopathy of grade 2 or higher, or infection within 1 month preceding the study or during the study -presence of diabetes, intrinsic renal or cardiovascular disease, or arterial hypertension on history and physical examination; abnormal urine analysis, chest radiograph, or electrocardiogram - presence of hepatocellular carcinoma or portal vein thrombosis - no treatment with drugs with known effects on systemic and renal hemodynamics within 7 days before inclusion"	-	Not specified.
Solà et al. 2018	Midodrine &Albumin For Preventing Complications In Patients With Cirrhosis Awaiting Liver transplantation	Spain	RCT	August 2008	March 2015	"Age older than 18 yr Cirrhosis defined by standard clinical, analytical and/or histological criteria Patients in the waiting list for liver transplantation Ascites Written informed consent."	"Arterial hypertension defined as systolic arterial pressure ≥150mmHg and/or diastolic arterial pressure ≥90mmHg or drug therapy for arterial hypertension Treatment with psychotropic drugs; transjugular intrahepatic portosystemic shunt (TIPS) Treatment with antibiotics within the last 7 days prior to study inclusion except for norfloxacin or rifaximin as prophylaxis for SBP or recurrent HE, respectively Chronic heart or respiratory failure; listed for combined liver-kidney transplant; previous liver transplant"	Not specified.
